# Asepsis and Antisepsis in Dentistry

**Published:** 1915-07

**Authors:** Andrew Kuehn, John A. Kimmel


### ASEPSIS AND ANTISEPSIS IN DENTISTRY.

READ BEFORE THE STUDENTS' DENTAL SOCIETY OF THE UNIVERSITY OF MICHIGAN, APRIL 20, 1915.

BY ANDREW KUEHN AND JOHN A. KIMMEL.

The subject for this paper was to have been "Asepsis in Dentistry," but as in operations about the mouth, aseptic surgery in itself is hardly possible, and the procedure is always a combination of the aseptic and antiseptic, we have taken the liberty of lengthening the title of our subject to "Asepsis and Antisepsis in Dentistry." The essential difference between antiseptic and aseptic surgery is that in the former the germs have supposedly gained entrance to the wound, and an effort is made to destroy them there by chemical antiseptics, while in the latter an endeavor is made to prevent their entrance to the wound. The aseptic method is a natural evolution of the antiseptic.

A dentist is called upon to perform surgical operations about the mouth which require scientific knowledge of the human body, and disease conditions. In 1878 Pasteur announced to the world that fermentation and putrefaction are due to the action of living organisms. Up to this time little attention had been given to the sterilizing of instruments or to the care of the field of operation. Instruments were cleaned after the operation by washing with soap and water and put away ready for future use, in the same way as the camper washes his dishes. The oldest coat was the proper thing to wear, and if the cuffs were turned up, it was to protect the coat, not the patient. Inflammation and suppuration were considered necessary accompaniments to wounds. The passing of the old time or septic surgery to aseptic or present day surgery has its beginning in 1865 when Joseph Lister of Glasgow published the results of his experiments and practically established the germ theory of disease. Lister demonstrated by experiment that a wound made in a clean skin by clean instruments and subsequently protected by clean dressings, would heal without inflammation or suppuration. He believed that the air was the principal carrier of germs, and hence impregnated the atmosphere of the operating room with a carbolic spray, which was made to play upon the hands of operator and field of operation constantly; instruments, dressings, etc., were soaked in the same. This is the wave of carbolic acid upon which Dr. Catell says he entered dentistry. Lister's proof that the suppuration of wounds was the result of microbic infection, was the great turning point in the history of surgery. His method of procedure still stands as a basis for modern aseptic surgery, and may be epitomized as follows:

1. Disinfection of the operator's hands, instruments, etc.

2. Purification of the wound and its environment.

3. Efforts to obviate the danger of atmospheric contagion.

4. Prevention of subsequent infection by suitable antiseptic dressings.

When later new discoveries were made by Robert Koch, improved methods of sterilization were introduced, such as sterilization by live heat and boiling water, and it was found that air was not the principal carrier of germs as supposed by Lister, but that the most common source of infection were the hands of the operator and the instruments, etc., used during the operation.

The keynote to modern surgical technic is surgical cleanliness, and this is best secured by a combination of the principles of asepsis and antisepsis. This should be the course followed in dentistry, and the nearer we get to aseptic treatment the nearer we are to perfection. Present day surgery demands that whatever antiseptic precaution is practicable should be used before the operation, and asepsis should be maintained during and after the operation, as it has been clearly demonstrated that chemicals introduced into a clean wound do no good, but much harm. The fact that today such marvelous results are accomplished in surgery, is mainly due to the aseptic precautions which are carried out. When, however, the wound is an accidental one, and not of the surgeon's making, it must be treated antiseptically. Free incision and thorough drainage which allow access of air are probably more important than irrigation with antiseptics. As dentists, we are convinced of the absolute necessity of precaution against all the dangers of sepsis. Apart from the surgical operations we may perform we should guard against all possible sources of infection, by having our clamps, separators, excavators, handpieces, etc., in a sterile condition before use. We are all aware of the septic conditions in the mouth which arise from improperly filled roots, badly fitting crowns, overhanging fillings, etc., results for which the dentist is responsible. So fatally true is it that "prevention is better than cure," that we ought to be sufficiently intelligent and sufficiently alert to the importance of the matter and carry out this aseptic teaching in practice.

With all of us there is a tendency to find an easy way of doing things, but let not the short cuts result in imperfect work and indifferent service to the public, laying septic traps, the injury from which exceeds by far the temporary good we do. The dental profession has in the past accused the medical profession of paying too little attention to the mouth for causes of disease, such as improper mastication, due to the irregularity or loss of teeth. Today, the growing tendency of the importance of pathological conditions in the mouth, and their relation to general health is awakening a respect for the dentist. When the medical man begins to make it a practice to examine the mouth, the greater becomes our responsibility, lest he finds there as the causes of disease, septic conditions which he suspects may be traced to dental incompetence.

